# Effect of nitric oxide deficiency on the pulmonary PTHrP system

**DOI:** 10.1111/jcmm.12942

**Published:** 2016-08-31

**Authors:** Bastian Brockhoff, Rolf Schreckenberg, Svenja Forst, Jacqueline Heger, Péter Bencsik, Krisztina Kiss, Peter Ferdinandy, Rainer Schulz, Klaus‐Dieter Schlüter

**Affiliations:** ^1^ Physiologisches InstitutJustus‐Liebig‐Universität GießenGießenGermany; ^2^ Pharmahungary GroupSzegedHungary; ^3^ Cardiovascular Research GroupDepartment of BiochemistryUniversity of SzegedSzegedHungary; ^4^ Department of Pharmacology and PharmacotherapySemmelweis UniversityBudapestHungary

**Keywords:** ADRP, PPARγ, elastin, lung fibrosis

## Abstract

Nitric oxide (NO) deficiency is common in pulmonary diseases, but its effect on pulmonary remodelling is still controversial. As pulmonary parathyroid hormone‐related protein (PTHrP) expression is a key regulator of pulmonary fibrosis and development, the effect of chronic NO deficiency on the pulmonary PTHrP system and its relationship with oxidative stress was addressed. NO bioavailability in adult rats was reduced by systemic administration of L‐NAME *via* tap water. To clarify the role of NO synthase (NOS)‐3‐derived NO on pulmonary expression of PTHrP, NOS‐3‐deficient mice were used. Captopril and hydralazine were used to reduce the hypertensive effect of L‐NAME treatment and to interfere with the pulmonary renin‐angiotensin system (RAS). Quantitative RT‐PCR and immunoblot techniques were used to characterize the expression of key proteins involved in pulmonary remodelling. L‐NAME administration significantly reduced pulmonary NO concentration and caused oxidative stress as characterized by increased pulmonary nitrite concentration and increased expression of NOX2, p47phox and p67phox. Furthermore, L‐NAME induced the pulmonary expression of PTHrP and of its corresponding receptor, PTH‐1R. Expression of PTHrP and PTH‐1R correlated with the expression of two well‐established PTHrP downstream targets, ADRP and PPARγ, suggesting an activation of the pulmonary PTHrP system by NO deficiency. Captopril reduced the expression of PTHrP, profibrotic markers and ornithine decarboxylase, but neither that of PTH‐1R nor that of ADRP and PPARγ. All transcriptional changes were confirmed in NOS‐3‐deficient mice. In conclusion, NOS‐3‐derived NO suppresses pulmonary PTHrP and PTH‐1R expression, thereby modifying pulmonary remodelling.

## Introduction

Nitric oxide (NO) is a widely expressed transmitter. In the lung, the main source of NO is endothelial NO synthase (eNOS=NOS‐3) that is expressed in endothelial and epithelial cells 1, 2, 3. Inhalation of low levels of NO dilates vessels within well‐ventilated regions of the lung indicating a physiological role for NO in pulmonary perfusion control. Inhaled NO was also found to normalize excessive elastin deposition as found in chronic lung disease 4. Endothelial dysfunction is associated with an inability of cells to release NO and it is found in many forms of pulmonary disease such as pulmonary arterial hypertension, chronic obstructive pulmonary disease, congestive heart failure and diabetes 5, 6, 7, 8, 9. However, excessive NO release is also contradictory to maintain a proper pulmonary function. In caveolin‐1‐deficient mice, a subsequent persistent activation of eNOS leads to nitration of protein kinase G, thereby inhibiting NO signalling pathways 10. Furthermore, NO decreases the expression of surfactant proteins in primary cultures of type II pneumocytes and in experimental lung transplantation 11, 12. As expected from these findings, NOS inhibition can restore pulmonary function under some conditions such as in hyperoxia‐induced lung injury in newborn rats and nickel‐induced acute lung injury 1, 13. Interestingly, inhibition of eNOS was found to restore surfactant gene expression 1 but a mechanistic link between eNOS‐derived NO deficiency and restored surfactant formation is lacking.

A central role in structural adaptation of the lung plays the expression of parathyroid hormone‐related protein (PTHrP). PTHrP binds to PTH‐1 receptors (PTH‐1R) and thereby it activates adenylyl cyclase and subsequently cAMP‐dependent protein kinase A. In the lung, this leads to an increased expression of peroxisome proliferator‐activated receptor (PPAR)‐γ, leptin, and adipose differentiation‐related protein (ADRP), an increased uptake of triglycerides, a prerequisite for surfactant formation, and finally attenuation of alveolar lipofibroblast‐to‐myofibroblast transdifferentiation 14. As expected from these previous findings, elevated PTHrP in epithelial lining fluid negatively correlates with the severity of lung injury in humans 15. Acute inhibition of NO formation reduces the secretion of surfactant proteins. However, high levels of NO reduced ATP‐induced surfactant secretion of type‐II alveolar cells 16. As both NO and PTHrP are related to surfactant and pulmonary structure formation, we suggested that NO affects the lung by interfering with PTHrP expression and PTH‐1R activation.

Reduced NOS activity is common in many forms of pulmonary disease and favours oxidative stress and pulmonary fibrosis 17, 18. The coupling between low NO level and oxidative stress is a complex interplay between radical production by uncoupling of NOS and activation of a local renin‐angiotensin system (RAS) 19, 20, 21. A RAS‐dependent activation of pulmonary transforming growth factor (TGF)‐β_1_ expression may couple RAS activation and fibrosis with oxidative stress 21. On the other hand, reduced levels of NO favour surfactant formation and attenuate pulmonary fibrosis 1, 14. Thus, it is highly unclear how reduced NO formation will affect pulmonary structure and remodelling and therefore it remains unclear how the NO system can be used to improve future therapy of life‐threatening lung diseases.

In this study, we used two different models of NO deficiency, Nω‐Nitro‐l‐arginine methyl ester (l‐NAME)‐treated rats and eNOS‐deficient mice, to study the effect of low NO formation on the activation of the pulmonary PTHrP system, alveolar lipofibroblast‐to‐myofibroblast transdifferentiation and oxidative stress. Subsequently, inhibition of the local RAS system by an angiotensin‐converting enzyme inhibitor, captopril and administration of a direct vasodilating drug, hydralazine, was used to separate effects linked to NO deficiency from haemodynamic vascular stress. Collectively our data will show a constitutive repressive role of eNOS‐derived NO on the activation of the pulmonary PTHrP system.

## Materials and methods

### Mice, rats and treatment protocols

L‐NAME treatment (30 mg/kg/day in tap water) has been used before to affect NO levels in rats 22. Captopril (30 mg/kg/day in tap water) or hydralazine (20 mg/kg/day in tap water) was administered as described 22, 23. L‐NAME was administered to rats for 2 weeks before either captopril or hydralazine were added with continuation of L‐NAME treatment. eNOS^−/−^ mice were purchased from Jackson Laboratory (strain Nos3^tm1Unc^). They were crossed with C57BL/6J mice and genotyped by tail biopsies with subsequent polymerase chain reaction (PCR) as described before 23, 24. All rats and mice underwent a weekly health scoring 25. To sacrifice the animals, rats and mice were anaesthetized with isoflurane prior to cervical dislocation and the lungs were quickly removed and stored in fluid nitrogen until analysis. The animal experiments were approved by the local authorities. Cardiac tissue derived from these animals has already been characterized 23.

### Blood pressure

For determination of blood pressure, rats or mice were set in a separate chamber and blood pressure (peak systolic blood pressure) was determined *via* the tail‐cuff method (TSE‐system, 209000 Series) as described before 22. Briefly, the mean of 10 consecutive readings was obtained for each animal at weekly intervals. Before the start of the study, the animals were adjusted to the experimental procedure over 1 week.

### Measurement of peroxynitrite

To estimate peroxynitrite formation in the kidney, we measured peroxynitrite marker nitrotyrosine by ELISA (components from Cayman Chemicals, Ann Arbor, Michigan, USA) as previously described 23. Briefly, lung tissue samples were pulverized in liquid nitrogen, sonicated in 4× homogenzation buffer and centrifuged. Supernatants were then incubated overnight with an anti‐nitrotyrosine rabbit IgG and nitrotyrosine acetylcholinesterase tracer in pre‐coated (mouse anti‐rabbit IgG) microplates flowed by development with Ellman's reagent. Protein concentration of the samples was measured by the bicinchoninic acid assay.

### Measurement of nitric oxide

Nitric oxide was determined in tissue samples by electron spin resonance technique as described before in detail 26. Briefly, diethyldithiocarbamate dissolved in distilled water was injected separately from FeSO_4_ and sodium citrate to avoid precipitation of Fe^2+^(DETC)_3_. Five minutes after treatment, lungs were isolated and freeze clamped. Approximately 100 mg of tissue was placed into quartz tubes and stored in liquid nitrogen until assayed for ESR spectra of NO‐Fe^2+^‐(DETC)_2_ complex. electron spin resonance (ESR) spectra were recorded with Bruker ECS 106 (Rheinstetten, Germany) spectrometer.

### qRT‐PCR

Total RNA was isolated from lungs using peqGold TriFast (peqlab; Biotechnology GmbH, Erlangen, Germany) according to the manufacturer's protocol as described before for heart tissues 22. To remove genomic DNA contamination, isolated RNA samples were treated with 1 U desoxyribonuclease per microgram RNA (Invitrogen, Karlsruhe, Germany) for 15 min. at 37°C. One microgram of total RNA was used in a 10‐μl reaction to synthesize cDNA using Superscript RNaseH reverse transcriptase (200 U/μg RNA; Invitrogen) and oligo dTs as primers. Real‐time quantitative PCR was performed with the Icycler IQ detection system (Bio‐Rad, Munich, Germany) in combination with IQ SYBR Green real‐time PCR supermix (Bio‐Rad). Primers used had the sequences according to Table 1. Quantification was performed as described before 27.

**Table 1:** List of primers used in this study

	Forward	Reverse
Rat		
HPRT	CCA GCG TCG TGA TTA GTG AT	CAA GTC TTT CAG TCC TGT CC
PTHrP	AGC TAC TCC GTG CCC TCC CG	AGG AAG AAA CGG CGG CGC AA
PTH‐1R	GGC TGC ACT GCA CGC GCA A	TTG CGC TTG AAG TCC AAC GC
ADRP	GCC CGA GTC ACA ACC CCA CG	AGA GTC GAC AGC CGC TCG GT
PPARγ	GCC GCA CGG ACG CAC ATT G	GCC TCA CAC GAC CCG GTA CC
Elastin	TGC TAC TGC TTG GTG GAG AAT G	CGT GGC TGC TGC TGT CTG
Collagen‐1	GCG AAC AAG GTG ACA GAG	CCA GGA GAA CCA GCA GAG
TGF‐β1	ATT CCT GGC GTT ACC TTG G	CCT GTA TTC CGT CTC CTT GG
ODC	GAA GAT GAG TCA AAC GAG CA	AGT AGA TGT TTG GCC TCT GG
Mouse		
HPRT	CCA GCG TCG TGA TTA GCG AT	CAA GTC TTT CAG TCC TGT CC
PTHrP	GAG ATC CAC ACA GCC GAA AT	CGT CTC CAC CTT GTT GGT TT
PTH‐1R	TTG CCT CCC TCA CCG TGG CT	CGG CGC GCA GCA TAA ACG AC
ADRP	CCC GCA ACC TGA CCC AGC AG	CGC CTG CCA TCA CCC CCA AG
PPARγ	GCC TTG CTG TGG GGA TGT	TCA GCG GGA AGG ACT TTA TGT
Elastin	CTG CTG CTA AGG CTG CTA AG	CCA CCA ACA CCA GGA ATG C
Collagen‐1	TTC TCC TGG RAA AGA TGG TGC	GGA CCA GCA TCA CCT TTA ACA
TGF‐β1	GTC CTT GCC CTC TAC AAC CA	GTT GGA CAA CTG CTC CAC CT
ODC	GAA GAT GAG TCA AAT GAA CA	AGT AGA TGT TTG GCC TCT GG

### Western blot

Tissue samples were incubated in lysis buffer as described before for cardiac tissue 22. Samples (100 μg) were loaded on a 12.5% SDS‐PAGE and blotted onto membranes as described before 22. Blots were incubated with a monoclonal antibody directed against PTHrP (antibody GF08; Calbiochem, Bad Soden, Germany) or PTH‐1 receptor (antibody 3D1.1; Upstate biotechnology, Eschborn, Gerrmany) as described before 28 and a second antibody coupled to horse radish peroxidase. Surfactant production was quantified by immunoblots detecting surfactant protein‐C (SP‐C; antibody FL‐197, sc‐13979 Santa Cruz Biotechnology) and lipofibroblast‐to‐myofibroblast transdifferentiation was analysed by up‐regulation of α‐smooth muscle protein (antibody clone 1A4, C6198 from Sigma‐Aldrich; St. Louis, USA).

### Statistics

Data are expressed as mean ± S.D. anova and, if indicated, Student–Neuman–Keuls test for *post hoc* analysis were used to analyse experiments in which more than one group was compared. Exact *P* values for anova testing is given in the figure legend or a *P* value of <0.05 compared to the respective control group is indicated by an asterisk. In cases where only two groups were compared, this was done by unpaired *t*‐test or Mann–Whitney test depending on the equal distribution of the samples as determined by Levene test.

## Results

### Effect of L‐NAME administration on pulmonary NO formation and oxidative stress

**Figure 1:**
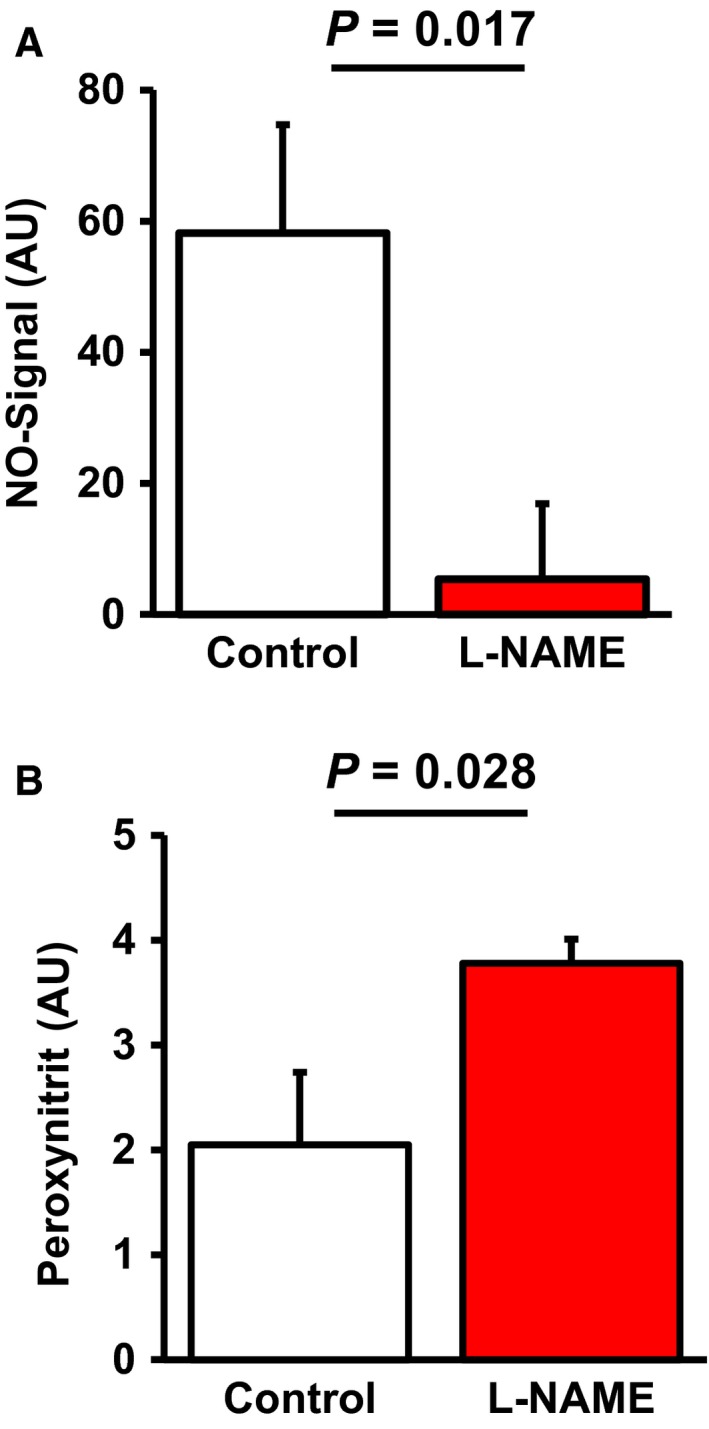
Effect of L‐NAME administration on pulmonary NO levels and nitrotyrosine content. Data are mean ± S.D. from *n* = 10 samples.

**Figure 2:**
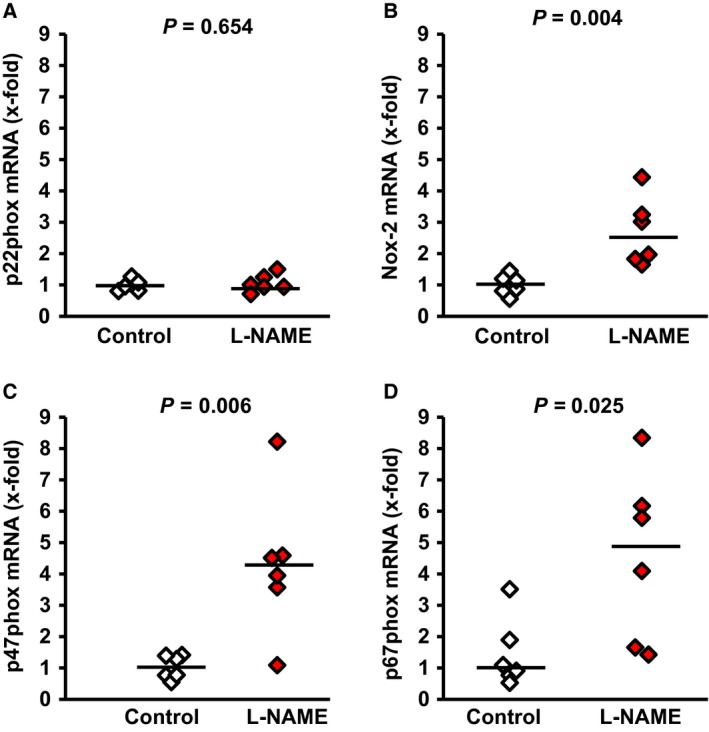
Effect of L‐NAME administration on the pulmonary mRNA expression of components of the NADPH oxidase complex. Data points represent the expression of the individual samples (*n* = 6). The bar indicates the median. Exact *P*‐values are given.

First, we proved that L‐NAME administration to rats successfully reduces pulmonary NO levels. Therefore, NO levels were quantified by ESR (Fig. 1A). The data show that L‐NAME significantly reduced NO formation by 90.7%. In parallel, reduced NO formation was accompanied by elevated pulmonary content of nitrotyrosin indicating elevated oxidative stress and functionally relevant modification of target proteins (Fig. 1B). Moreover, L‐NAME administration led to a significant up‐regulation of enzymes involved in the formation of reactive oxidative species (ROS), such as NOX2, p47phox and p67phox (Fig.2). Finally, L‐NAME administration induced systemic hypertension (Table 2). Collectively, these data confirmed known effects of NO deficiency and indicate the effectiveness of the protocol‐NAME dosage used here.

**Table 2:** Effect of L‐NAME on blood pressure

	*t* = 0	*t* = 6	*t* = 8	
Control	119 ± 8	121 ± 11	126 ± 16	*n* = 9–10
L‐NAME	120 ± 15	150 ± 15*	157 ± 22	*n* = 8–10
L‐NAME+Cap	117 ± 11	163 ± 18*	139 ± 10#	*n* = 10
L‐NAME+Hydra	126 ± 6	152 ± 13*	133 ± 12#	*n* = 10

Data are mean ± S.D. of systolic blood pressures in mmHg. *, *P* < 0.05 *vs*. *t* = 0; #, *P* < 0.05 *vs*. *t* = 6. Time‐points are *t* = 0 (start of the L‐NAME treatment), *t* = 6 (6 weeks after L‐NAME administration), and *t* = 8 (8 weeks after administration of L‐NAME with 2 week treatment with captopril and hydralazine.

### Effect of NO deficiency on the PTHrP system

**Figure 3:**
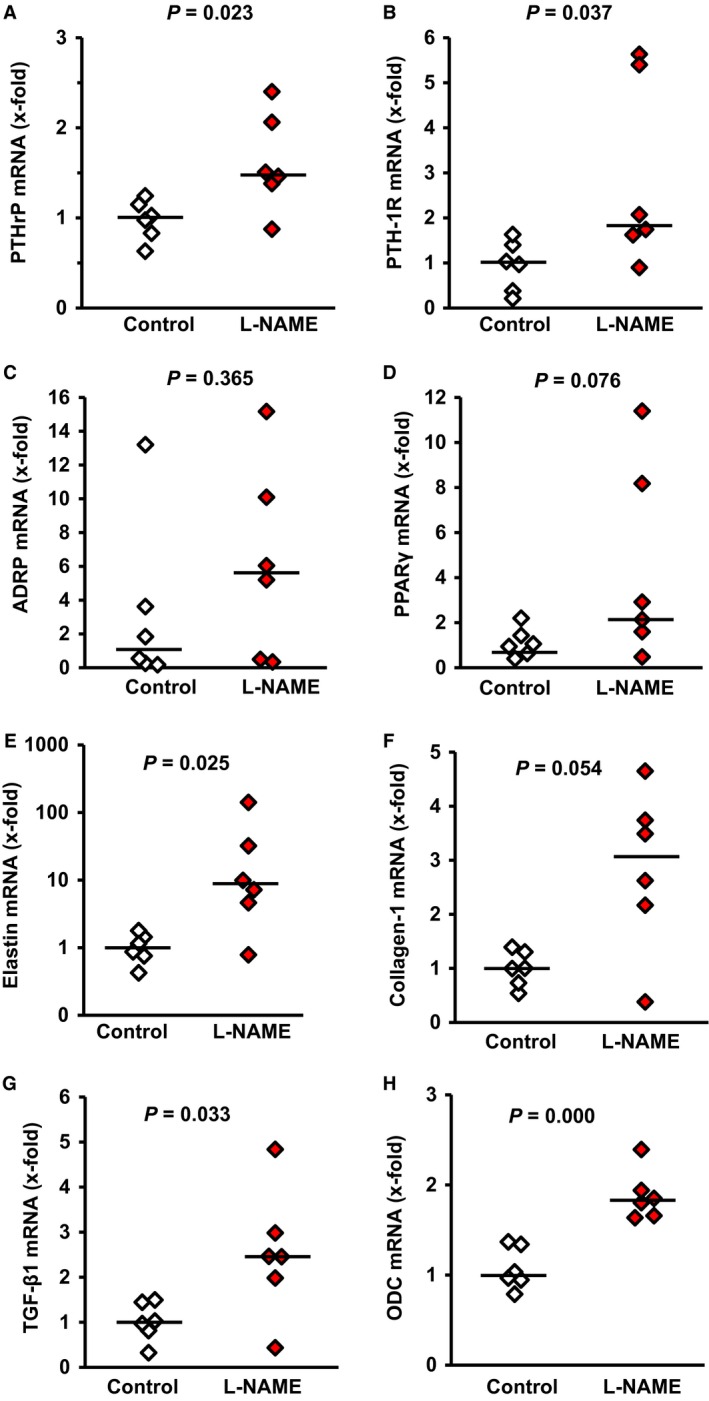
Effect of L‐NAME administration on the pulmonary mRNA expression of PTHrP, PTH‐1 receptor, and PTHrP‐downstream targets ADRP and PPARγ, and of fibrotic markers. Data points represent the expression of the individual samples (*n* = 6). The bar indicates the median. Exact *P* values are given.

**Figure 4:**
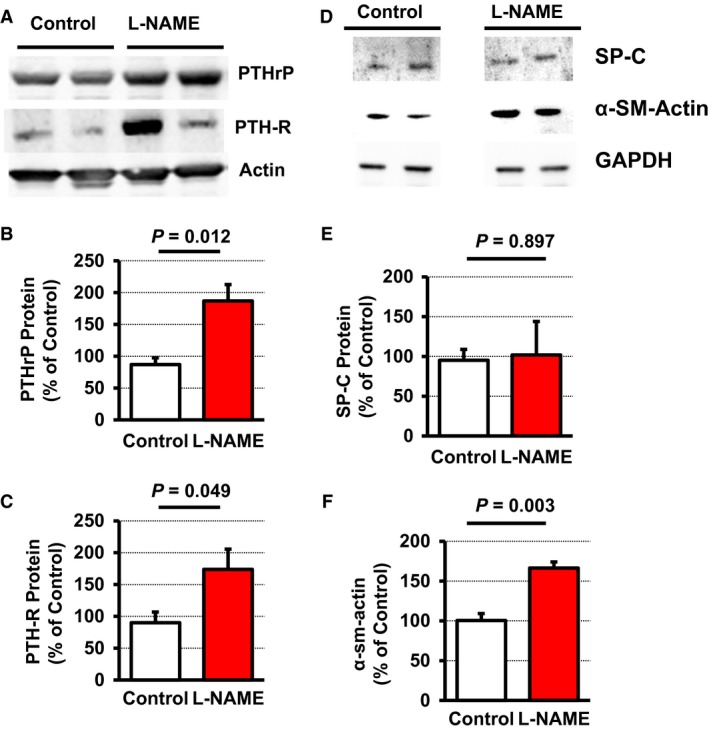
Effect of L‐NAME administration on the pulmonary protein expression of PTHrP, PTH‐1 receptor (PTH‐R), surfactant protein C (SP‐C), and α‐smooth muscle actin. Representative immunoblot is shown (**A**,** D**); Data are mean ± S.E.M. from *n* = 4–6 samples.

Once we have established that L‐NAME administration significantly affected pulmonary NO formation, we investigated next, whether L‐NAME administration modifies the expression of PTHrP and PTH‐1R in the lung. L‐NAME significantly increased the pulmonary expression of both proteins (Fig. 3A–D). Furthermore, pulmonary expression of PTHrP mRNA and PTH‐1R mRNA strongly correlated with the mRNA expression of its downstream target genes ADRP and PPARγ (*P* = 0.017, *r* ^2^ = 0.233 and *P* = 0.006, *r* ^2^ = 0.433 respectively). Similarly, mean expression of ADRP and PPARγ were increased in L‐NAME‐treated rats with elevated PTHrP and PTH‐1R expression, although the large variation suppressed *P* values below 0.05 (Fig. 3). More importantly, L‐NAME‐induced increase of the mRNA expression of PTHrP and of its corresponding receptor was confirmed on the protein level (Fig. 4A–C). An activation of the PTHrP system has been linked initially to surfactant production 14. However, PTH‐1R null mice had no difference in the expression of surfactant proteins, such as surfactant protein (SP)‐C 29. In line with the latter findings, we did not find differences in the expression of SP‐C (Fig. 4D,E).

As the PTHrP system attenuates an alveolar lipofibroblast‐to‐myofibroblast transdifferentiation, an initial step of pulmonary fibrosis, we expected that L‐NAME‐induced PTHrP expression will repress elastin expression and therefore pulmonary fibrosis. However, in L‐NAME‐treated rats elastin, collagen‐1 and TGF‐β_1_ were all up‐regulated as well as ornithine decarboxylase (ODC), a marker of cell growth (Fig. 3E–H). This suggests an onset of pulmonary fibrosis and this was confirmed on the protein level because the concentration of α‐smooth muscle actin increased (Fig. 4D,F).

### Effect of captopril and hydralazine on L‐NAME‐induced changes

**Figure 5:**
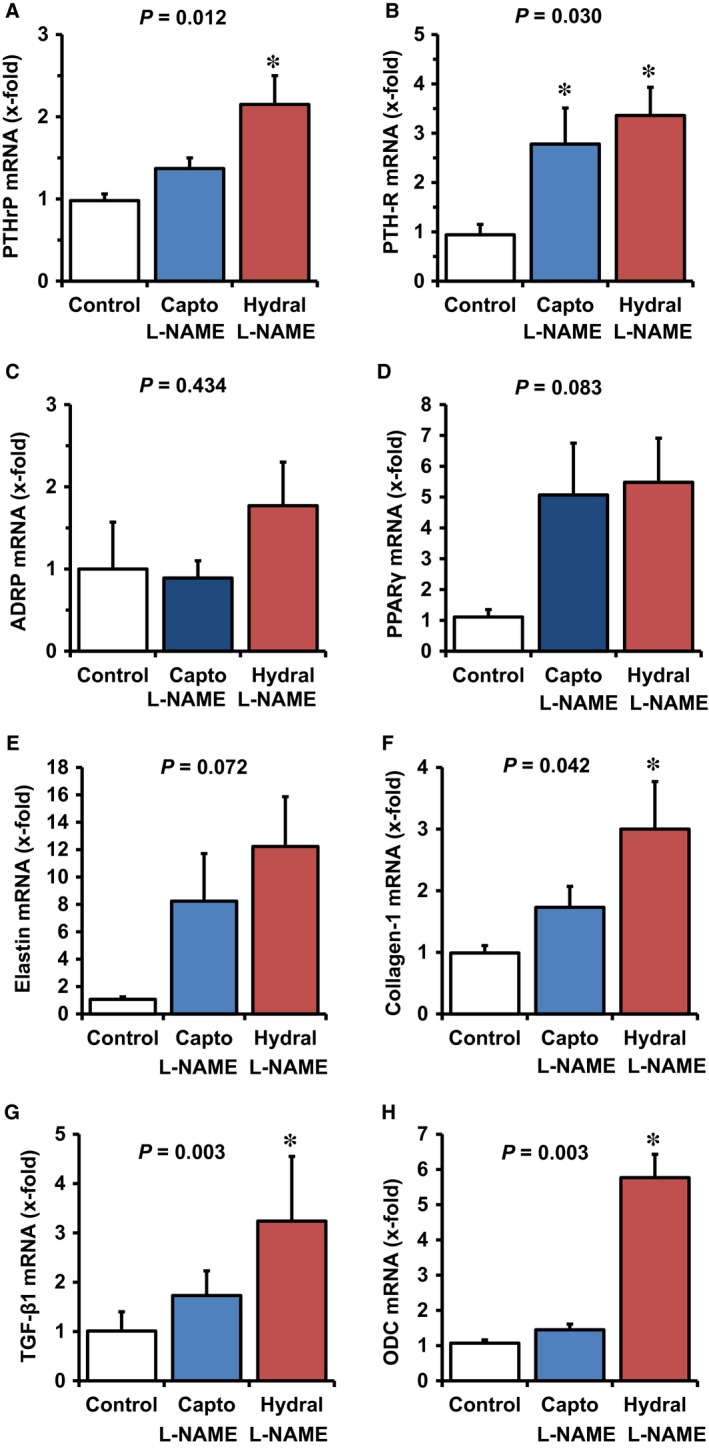
Effect of captopril and hydralazine on L‐NAME‐induced changes in the pulmonary mRNA expression of PTHrP, PTH‐1 receptor, and PTHrP downstream targets ADRP and PPARγ and that of fibrotic markers. Data are mean ± S.D. from *n* = 6 samples. Exact *P* values are given *, *P* < 0.05 *vs*. control.

The large variation in ADRP and PPARγ expression suggests co‐regulation by further factors. L‐NAME treatment is associated with an activation of RAS and with hypertension. Therefore, participation of RAS activation and hypertension in the aforementioned changes in PTHrP expression were investigated next. In the presence of captopril and hydralazine, blood pressure dropped (Table 2). Captopril normalized the expression of PTHrP and ADRP, but not that of PTH‐1R and PPARγ. Reducing the blood pressure by administration of hydralazine was less effective than inhibiting the RAS (Fig. 5A–D).

Pulmonary fibrosis, as indicated by up‐regulation of elastin, collagen‐1, TGF‐β_1_ and ODC, was attenuated by captopril but not by hydralazine. This suggests that pulmonary fibrosis is triggered by the activation of RAS rather than by high blood pressure (Fig. 5E–H).

### Pulmonary PTHrP expression in eNOS‐deficient mice

**Figure 6:**
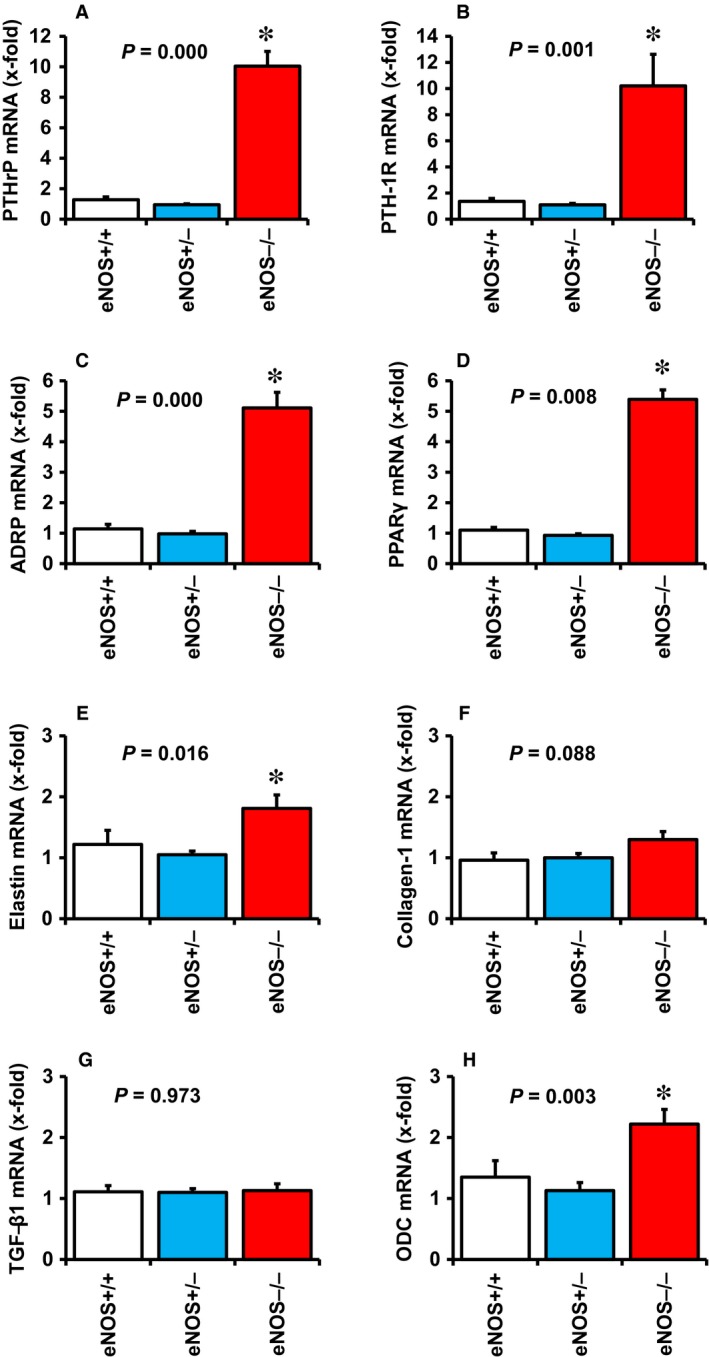
Effect of eNOS deficiency on pulmonary expression of PTHrP (**A**), PTH‐1 receptor (**B**), ADRP (**C**), PPARγ (**D**), elastin (**E**); collagen‐1 (**F**), TGF‐β1 (**G**), and ODC (**H**) in wild‐type mice (+/+), heterozygous mice (±) and eNOS knockout mice (−/−). *, *P* < 0.05 *vs*. eNOS +/+ Data are mean ± S.D. from *n* = 9–23 mice.

The aforementioned results suggest that pulmonary NO significantly controls the expression of PTHrP and thereby also of its down‐stream targets ADRP and PPARγ. As eNOS‐derived NO is the main source of pulmonary NO, we finally investigated whether the above mentioned changes are depending on eNOS‐derived NO. Pulmonary expression of PTHrP, PTH‐1R, ADRP and PPARγ were all significantly elevated in eNOS‐deficient mice (Fig. 6A–D). In contrast, the pulmonary expression of fibrotic markers was less significantly affected by eNOS deficiency and only small increases in elastin and ODC were found (Fig. 6E–H).

## Discussion

This study evaluates the role of NO for pulmonary expression and activity of the PTHrP system. In the lung, PTHrP is expected to increase surfactant production and to reduce lipofibroblast‐to‐myofibroblast transdifferentiation 14. The main finding of this study is that NO deficiency directly increases the pulmonary expression of PTHrP and of PTH‐1R. Of note, this effect was similarly found in L‐NAME‐treated rats and eNOS‐deficient mice. Therefore, the data of this study identify eNOS‐derived NO as an endogenous inhibitor of the pulmonary PTHrP system. Furthermore, the data of this study show that NO represses the pulmonary expression of ADRP and PPARγ. Both proteins are regulated in a PTHrP‐dependent way. *In vitro* findings suggested that high NO levels suppress surfactant secretion and vice versa 16. This process depends on the activation of the PTHrP system. Here, we observed the opposite effect and therefore forced the view that NO suppresses surfactant formation but lowering NO levels increases the PTHrP pathway. miR33 has been reported to depress PTHrP expression and may be a candidate that triggers this effects 29. A link between NO/cGMP and miR33, however, is still missing. A direct coupling of increased expression of PTHrP and its down‐stream targets PPARγ and ADRP to surfactant formation would require an increased expression of SP‐C, but this was not found. In agreement with these findings, studies with PTH‐1R null mice have already shown that PTHrP may not be linked to lung development as receptor deficiency does not affect major peripheral proteins of the lung 30.

A dysfunctional pulmonary NO system has been implicated in many forms of lung disease. This might be expected because pulmonary epithelial and endothelial cells express the same constitutive isoform of NOS, namely eNOS. Interestingly, while inhaled NO and stimulation of downstream targets of NO, such as soluble guanylyl cyclase activators, suppress some forms of lung disease and inhibit pulmonary fibrosis, blockade of NO formation was found to be beneficial in other conditions 31. Thus, the role of NO in adaptation of pulmonary structure and function under stress conditions seems to be very complex in nature. Our study confirmed earlier reports about an effect of L‐NAME on pulmonary fibrosis. In NO‐deficient mice and L‐NAME‐treated rats, pulmonary expressions of fibrotic markers were collectively up‐regulated under hypobaric hypoxia 17, 32. The expression of elastin and collagen‐1 were no longer increased when L‐NAME was administered in the presence of captopril. In the presence of captopril, the level of TGF‐β_1_ expression was at least lower than that induced by L‐NAME alone. In summary, this part of the study suggests that L‐NAME causes pulmonary fibrosis *via* an activation of RAS. The lung has the highest angiotensin‐converting enzyme (ACE) activity compared to aorta, heart or kidney. However, in contrast to the aforementioned tissues, L‐NAME does not further increase its already high activity 19. Therefore, we suggest that activation of RAS upstream of ACE triggers captopril‐sensitive effects of L‐NAME on pulmonary remodelling. In line with these assumptions, transgenic (mRen2)27 rats display increased expression of components of NOX and a similar increase was obtained here in the tissue samples of L‐NAME‐treated rats 21. The conclusion that RAS rather than haemodynamic load causes the observed pulmonary fibrosis is supported by our finding that hydralazine did not cause a similar effect as captopril although it had a clear vasodilating effect.

In principle, RAS‐dependent activation of TGF‐β may also directly increase the expression of PTHrP in the lung. TGF‐β_1_ induces the wnt signalling in osteolytic breast cancer cells and up‐regulates PTHrP 33. However, it is unlikely that in the absence of NO, the wnt pathway affects pulmonary expression of PTHrP. First, in eNOS‐deficient mice, PTHrP expression is strongly increased in the absence of increased expression of TGF‐β_1_. Second, in rats captopril attenuated the expression of TGF‐β_1_ but neither that of PTH‐1R nor that of PPARγ. Furthermore, nicotine induces the wnt pathway, but decreases the expression of PTHrP and PPARγ 34. Therefore, the role of the wnt pathway in pulmonary expression of PTHrP has to be established in the future. Alternatively, activation of the Notch pathway has been linked to epithelial‐mesenchymal transition. However, although *in vitro* studies with breast cancer cells suggest a Notch‐dependent co‐regulation of PTHrP pathways and epithelial‐mesenchymal transition 33, our own experiments *in vivo* with NO deficiency suggest a different regulation of these pathways. This makes it unlikely that Notch activation plays a relevant role.

In this study we used the expression of elastin as a marker of pulmonary fibrosis because excessive elastin deposition characterizes chronic lung disease 4. Furthermore, we analysed the pulmonary expression of collagen‐1 and TGF‐β_1_ because epithelial‐mesenchymal transition is favoured by TGF‐β_1_‐ and TGF‐β_1_‐dependent collagen‐1 synthesis but reciprocally expressed in the presence of NO 35. Epithelial‐mesenchymal transition was further confirmed by increased α‐smooth muscle protein. Finally, we analysed the expression of pulmonary ornithine decarboxylase (ODC) because this rate‐limiting enzyme of the polyamine metabolism is required for pulmonary tissue growth and remodelling 36. In our study, NO deficiency affected the PTHrP system and the RAS‐TGF‐β_1_ cascade in a different way. Therefore, we conclude that the pulmonary PTHrP system seems more closely linked to ADRP and PPARγ rather than to inhibition of pulmonary fibrosis. This does not mean that an activated PTHrP system does not attenuate the alveolar transepithelial‐to‐myofibroblast transdifferentiation, as suggested before, but in the context of NO deficiency its main effect is on ADRP and PPARγ expression rather than on fibrosis. At the same time, an activation of RAS overrides a potential anti‐fibrotic effect of PTHrP. From a therapeutic point of view this leads to a dilemma: Although NO deficiency increases PTHrP expression and thereby ADRP and PPARγ expression, the same trigger favours pulmonary fibrosis. An activation of RAS and RAS‐dependent oxidative stress may support this consequence of NO deficiency as this went alongside with increased expression of NOX2, p47phox and p67phox. Indeed, we found not only a significant drop in pulmonary NO, as determined by direct measurement of NO by ESR but also an increase in tissue nitrotyrosine, a marker of oxidative stress relative to NO.

**Figure 7:**
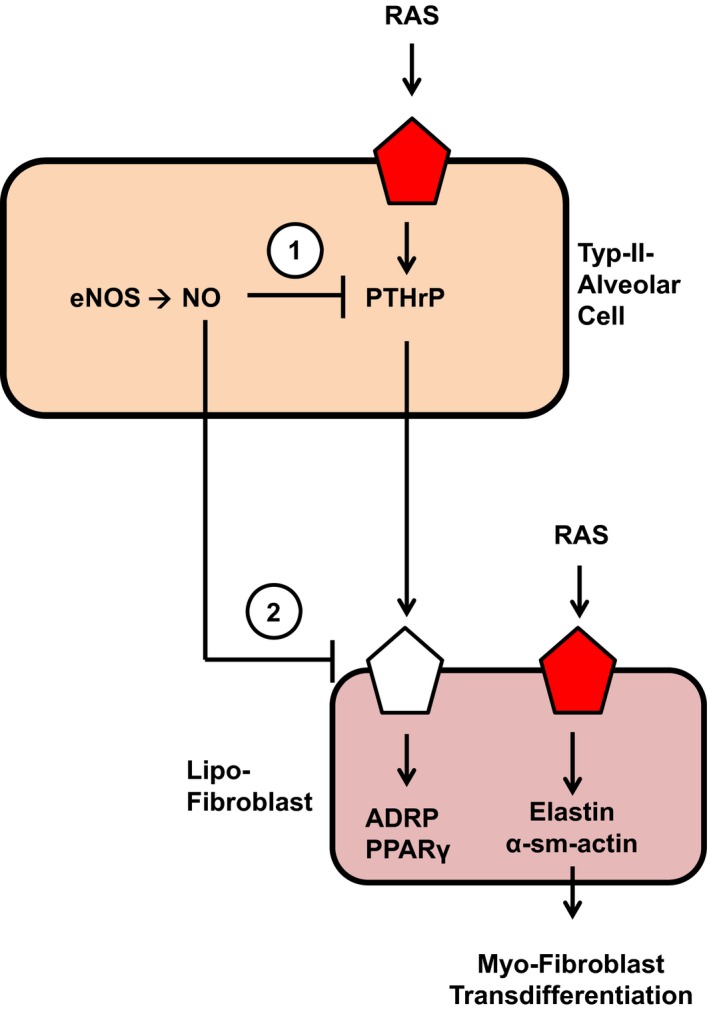
Conclusive summary of the data obtained in this study. NO constitutively represses the pulmonary expression of PTHrP in alveolar type II cells (1) and PTH‐1 receptors (2) in lipofibroblasts, thereby controlling their expression of ADRP and PPARγ, two proteins required for proper formation of surfactant. RAS seems to modify pulmonary fibrosis independent of a potential role of PTHrP for the transition of alveolar lipofibroblasts to myofibroblasts.

In summary we came to the following view as indicated in Fig. 7: NO constitutively represses pulmonary PTHrP and PTH‐1R expression thereby keeping expression of ADRP and PPARγ rather low. NO deficiency and the lack of soluble guanylyl cyclase stimulation activates RAS that then triggers pulmonary fibrosis most probably by its ROS‐forming effect. At the same time, pulmonary PTHrP is induced but not sufficient to compensate the pro‐fibrotic effect of RAS activation.

## Conflict of interest

The authors declare that they have no competing interest.
